# Draft Genome Sequences of Facultative Methylotrophs, *Gemmobacter* sp. Strain LW1 and *Mesorhizobium* sp. Strain 1M-11, Isolated from Movile Cave, Romania

**DOI:** 10.1128/genomeA.01266-15

**Published:** 2015-11-19

**Authors:** Deepak Kumaresan, Daniela Wischer, Alexandra M. Hillebrand-Voiculescu, J. Colin Murrell

**Affiliations:** a School of Environmental Sciences, University of East Anglia, Norwich, United Kingdom; b Emil Racoviţă Institute of Speleology, Bucharest, Romania; c School of Earth and Environment, The University of Western Australia, Perth, Australia

## Abstract

Facultative methylotrophs belonging to the genera *Gemmobacter* and *Mesorhizobium* were isolated from microbial mat and cave water samples obtained from the Movile Cave ecosystem. Both bacteria can utilize methylated amines as their sole carbon and nitrogen source. Here, we report the draft genome sequences of *Gemmobacter* sp. strain LW1 and *Mesorhizobium* sp. strain IM1.

## GENOME ANNOUNCEMENT

Movile Cave (Mangalia, Romania) is a hypogenic cave ecosystem that has been isolated from the surface for 5.5 million years and is devoid of any input of organic carbon from above (1). Invertebrates present in the cave are adapted to life in the dark and are supported by chemolithoautotrophic primary producers that derive energy from the oxidation of inorganic compounds (hydrogen sulfide, hydrogen, and methane) (2, 3). Degradation of the microbial mats floating on the surface of the cave water probably produces large amounts of methylated amines (MA), as indicated by the apparent abundance and activity of MA degraders (4, 5). Here, we report the draft genome sequences of two facultative methylotrophs, *Gemmobacter* sp. strain LW1 and *Mesorhizobium* sp. strain 1M-11, isolated from cave water and a microbial mat, respectively (5). DNA from the isolates was obtained using the phenol-chloroform method (6). The draft genome sequences were generated at The Genome Analysis Centre (TGAC), Norwich, United Kingdom, using the Illumina platform. The raw sequences were assembled using ABySS version 1.3.4 (7) using a range of *k*-mer sizes. The best-performing assembly (*k*-mer-wise and filtered versus unfiltered) was selected based on the assembly metrics and was subsequently scaffolded further using SSPACE version 2.0 (8). GapCloser-1.12 was then used to close any gaps in the scaffolded assembly. All reads were quality trimmed using Sickle version 1.1 (GitHub) based on a Q30 quality score. Genome annotation was performed using the RAST annotation server (9).

*Gemmobacter* sp. LW1 belongs to the family *Rhodobacteraceae*, and the genus *Gemmobacter* includes only five validated species, which were recently reassigned from the genus *Catellibacterium* (10). The genome includes 4,256 coding sequences (CDSs) and 79 tRNAs, and it is 4.35 Mb in size. *Mesorhizobium* sp. 1M-11 (family *Phyllobacteriaceae*; 6,592 CDSs, 79 tRNAs, and 6.69 Mb in size), closely related to *Mesorhizobium loti*, based on 16S rRNA gene sequence identity (11), is the only known member of the genus *Mesorhizobium* to grow methylotrophically. Even though *M. loti* possesses genes (i.e., *gmaS*) involved in the *N*-methylglutamate pathway, this organism cannot grow methylotrophically on methylated amines (12). The gene clusters responsible for methylamine utilization, through both methylamine dehydrogenase (13) and *N*-methylglutamate pathways (14, 15), were identified in the genomes of both isolates. Also, genes encoding the enzyme trimethylamine monooxygenase (Tmm) (16) are present in both the genomes, with the metabolic potential confirmed by growth on trimethylamine as the sole carbon and nitrogen source (5). Genes encoding enzymes of the pentose phosphate pathway, Entner-Doudoroff (a variant of the ribulose monophosphate [RuMP] pathway) pathway, the tricarboxylic acid (TCA), and serine cycles were also predicted. The gene *folD*, encoding the enzyme 5,10 methylene-tetrahydrofolate dehydrogenase/cyclohydrolase, is present in these genomes, suggesting that formaldehyde is utilized through tetrahydrofolate (H_4_F) (genes encoding key enzymes in the tetrahydromethanopterin [H_4_MPT]-mediated C_1_ oxidation pathway are absent) (17). While genes coding for sulfur oxidation pathways are present in both isolate genomes, genes involved in denitrification (*nirS*-type), propane (*prmA*), and carbon monoxide (*coxL*) oxidation were predicted only in the genome of *Gemmobacter* sp. LW1. In summary, these genome sequences present a metabolic blueprint for these two methylotrophic isolates from Movile Cave, and they provide excellent model organisms for understanding methylotrophy in this unusual ecosystem.

### Nucleotide sequences accession numbers.

This whole-genome shotgun project has been deposited at GenBank under the accession numbers LJSC00000000 and LJSD00000000. The versions described in this paper are versions LJSC01000000 and LJSD01000000.
